# Maximum likelihood based analysis of equally spaced longitudinal count data with first-order antedependence and overdispersion

**DOI:** 10.1186/s40064-016-3564-8

**Published:** 2016-11-08

**Authors:** Victoria Gamerman, Matthew Guerra, Justine Shults

**Affiliations:** 1 Boehringer-Ingelheim Pharmaceuticals, Inc., 900 Ridgebury Road, Ridgefield, CT 06877 USA; 2 Division of Biometrics III, OB, OTS, CDER, FDA, Silver Spring, MD 20993 USA; 3 Department of Biostatistics, University of Pennsylvania, 423 Guardian Drive, Philadelphia, PA 19104 USA

**Keywords:** Count data, First-order antedependence, Generalized estimating equations, Markov property, Maximum likelihood estimation, Over-dispersion

## Abstract

This manuscript implements a maximum likelihood based approach that is appropriate for equally spaced longitudinal count data with over-dispersion, so that the variance of the outcome variable is larger than expected for the assumed Poisson distribution. We implement the proposed method in the analysis of seizure data and a subset of German Socio-Economic Panel data. To demonstrate the importance of correctly modeling the over-dispersion, we make comparisons with the semi-parametric generalized estimating equations approach that incorrectly ignores any over-dispersion in the data. Our simulations demonstrate that accounting for over-dispersion results in improved small-sample efficiency and appropriate coverage probabilities. We also provide code in R so that readers can implement our approach in their own analyses.

## Background

Longitudinal count data are often encountered in scientific studies. For example, Thall and Vail (1990) analyzed repeated seizure counts on subjects in a clinical trial. Winkelmann (2004) analyzed doctor visits, to evaluate whether German health care reform caused a change in their distribution.

Common features of serial count data include intra-subject correlation, due to similarity between the repeated measurements on each participant, and over-dispersion, which occurs when the variance is larger than expected for the assumed distribution of the outcome variable (Efron 1992). Poisson regression is often applied for analysis of count data, but is usually not appropriate for longitudinal studies because it ignores intra-subject correlations and over-dispersion. Generalized Poisson regression (Consul and Famoye 1992) allows for both over- and under- dispersion, but assumes independence of measurements.

In this paper we implement a maximum-likelihood based method for the analysis of longitudinal count data with over-dispersion induced by the serial correlation of measurements. Key assumptions of the approach include the first-order Markov property and linearity of the expectations for the conditional distributions, which are assumed to be Poisson. In addition, we assume that the correlation between adjacent measurements on a subject is constant.

The assumptions of the first-order Markov property, linearity in the conditional expectations, and constant adjacent correlations have been shown to induce a first-order autoregressive AR(1) correlation structure for the repeated outcomes on each subject (Guerra and Shults 2014). The AR(1) structure forces a decline in the intra-subject correlations with increasing separation in time. Our method is therefore most appropriate for analysis of equally spaced longitudinal count data with over-dispersion.

Other approaches for analysis of over-dispersed longitudinal count data include semi-parametric approaches such as generalized estimating equations (GEE) (Liang and Zeger 1986). Vinod (2002) described econometric applications of GEE. Ghisletta and Spini (2004) provided a concise summary of GEE for the social sciences. GEE is widely used because it does not require specification of the full likelihood that can be quite complex for longitudinal discrete data. However, GEE does not account for over-dispersion. In addition, the relative ease of application of GEE for discrete data can also be a potential limitation for the approach. When only the first two moments of the distribution of the outcome variable are estimated, as they are for GEE, it is possible to obtain estimates that are not compatible with any valid parent distribution. As cautioned by Molenberghs and Kenward (2010), “the parent provides a natural description of the framework into which the semi-parametrically specified parameters fit. The implication is that such semi-parametric methods as GEE1, GEE2, ALR, etc. can always be applied because there is always a valid parent, and hence a probabilistic basis.”

We make comparisons with GEE to evaluate the impact of incorrectly ignoring over-dispersion when the models for the marginal mean and correlation structure are correct. We conduct simulations for moderately sized samples to demonstrate that when the likelihood is correctly specified, we have improved efficiency in estimation of the regression and correlation parameters for our approach relative to GEE that incorrectly ignores the over-dispersion.

Another model for longitudinal count data is the class of generalized linear mixed-effects models that incorporate random effects in the linear predictor. However, the implementation of likelihood based methods that involve random effects can be computationally challenging (p. 75, Fitzmaurice et al. 2008). In addition, in contrast to GEE, for mixed models it is not straightforward to specify a particular working correlation structure for the repeated measurements on subjects. For example, the AR(1) correlation structure is not among the covariance models that were suggested by Thall and Vail (1990). Mixed-effects models are typically employed when the goal is to estimate effects that are subject specific, because the analysis results are conditional on the random effects (Gardiner et al. 2009).

In general, likelihood based approaches like the one we implement in this paper enjoy several general advantages. Unlike semi-parametric approaches, they yield an estimated likelihood that can be used to conduct likelihood ratio tests and to compare the fit of models using criteria such as the Akaike information criterion (AIC) (Akaike 1974) and Bayesian information criterion (BIC) (Schwarz 1978). Maximum likelihood estimators are also most (asymptotically) efficient among a wide class of estimators (Serfling 2011) when the distribution is correctly specified. Our method in particular, allows for specification of the usual model for the marginal mean for Poisson data, while also accounting for over-dispersion and serial correlation in the data via the induced AR(1) correlation structure.

In "Methods" section we discuss the notation, model assumptions, the likelihood and likelihood equations. In 'Application" section we discuss an application of the methods followed by simulation studies in "Simulation studies" section. We conclude with a discussion in Conclusion" section.

## Methods

### Notation and model assumptions

The data comprise realizations $y_{ij}$ of ordered discrete random variables $Y_{ij}$ that are measured on subject *i* at time $t_{ij}$ ($i = 1,\ldots ,m$ and $j = 1,\ldots ,n_i$). Associated with each $y_{ij}$ is a vector of explanatory variables (covariates) $x_{ij}=(x_{ij1},\ldots ,x_{ijp})^{'}$. The expected value of measurement $Y_{ij}$ on subject *i* is given by

1 $$\begin{aligned} E\left( Y_{ij}\right) =\mu _{ij} = \lambda _{ij}, \end{aligned}$$

and the variance by $\text{ var }(Y_{ij}) = \sigma ^2_{ij}.$

We assume that observations on different subjects are independent. Further, the measurements within subjects are correlated with a structure that depends on parameter $\alpha$. Let $\text{ cov }(Y_{ij},Y_{ik})$ represent the covariance and $\text{ corr }(Y_{ij},Y_{ik})$ represent the correlation between $Y_{ij}$ and $Y_{ik}.$

We make three assumptions. First, we assume first-order antedependence, such that each $Y_{ij}$, given the immediate antecedent $Y_{ij-1}$, is independent of all further preceding variables (Gabriel 1962). The joint probability mass function of $Y_{i1},\ldots , Y_{in_i}$ can then be expressed as

2 $$ \text{P} \left(Y_{i1} =y_{i1},Y_{i2}=y_{i2}, \ldots ,Y_{in_i}=y_{in_i}\right) = \text{P} \left(Y_{i1}=y_{i1}\right) \text{P} \left(Y_{i2}=y_{i2}| Y_{i1}=y_{i1} \right) \cdots \text{P} \left(Y_{in_i}=y_{in_i}| Y_{i{n_i}-1}=y_{i{n_i}-1}\right) . $$

First-order antedependence is also referred to as the first-order Markov property in the literature (Feller 1968, p. 419).

Second, we assume that the correlation between adjacent measurements on a subject is constant, implying that

$$\begin{aligned} \text{ corr }\left( Y_{ij},Y_{ij-1}\right) = \alpha \end{aligned}$$

where $i=1\ldots ,m$ and $j=2,\ldots ,n_i$. Third, we assume that the conditional expectation of $Y_{ij}$ given $Y_{ij-1}$ is a linear function of $Y_{ij-1},$ such that

$$\begin{aligned} \text{ E }\left( Y_{ij} \mid Y_{ij-1}\right) = a_{ij} + b_{ij} Y_{ij-1}, \end{aligned}$$

for $$i=1\ldots ,m$$ and $$j=2,\ldots ,n_i$$.

These three assumptions imply the following results. From Theorem 2.1 of Guerra and Shults (2014), the conditional expectation is given by

3 $$\begin{aligned} \text{ E }\left( Y_{ij} | Y_{ij-1} \right) = \mu _{ij} + \alpha \sigma _{ij}/\sigma _{ij-1}\left( Y_{ij-1} - \mu _{ij-1}\right) , \end{aligned}$$

where $$\mu _{ij} = \text{ E }\left( Y_{ij}\right) ,$$ $$\alpha = \text{ corr }\left( Y_{ij-1},Y_{ij}\right) ,$$ $${\sigma _{ij}}^2 = \text{ var }\left( Y_{ij}\right) $$, and

4 $$\begin{aligned} {\sigma _{ij}}^2= & {} \frac{1}{1 - {\alpha }^2} \text{ E } \left( \text{ var } \left( Y_{ij}\, | \,Y_{ij-1}\right) \right) , \end{aligned}$$

where $$i=1,\ldots ,m$$ and $$j=2,\ldots ,n_i$$.

Next, from Section 2.5 of Zimmerman and Nuñez-Antón (2010), the correlation $\text{ corr }(Y_{ij},Y_{ij+t})$ between $Y_{ij}$ and $Y_{ij+t}$ for $t>0$ can be expressed as

$$\begin{aligned} \text{ corr }\left( Y_{ij},Y_{ij+t}\right) &= \prod _{k=j}^{j+t-1} \text{ corr }\left( Y_{ij},Y_{ij+1}\right) \\ &= \prod _{k=j}^{j+t-1} \alpha \\ & = \alpha ^{t}. \end{aligned}$$

The induced correlation structure for $\left( Y_{i1},\ldots ,Y_{in_i} \right) ^\prime$ is therefore an AR(1) structure.

This AR(1) structure is plausible for longitudinal data because it requires the correlation between measurements on a subject to decline with increasing separation in time. For example, if $\alpha = 0.5$, then the correlation between the 1st and 2nd measurements is 0.5, while the correlation between 1st and 3rd measurements is $(0.5)^2 = 0.25$.

### Poisson likelihood

We assume Poisson distributions for the marginal and conditional distributions in Eq. 2. For each $i=1,\ldots ,m$, the distribution of $Y_{i1}$ is Poisson with $\mu _{i1}$ = $\lambda _{i1}$ = $\text{ exp }\left( x_{i1}^\prime \beta \right)$ and ${\sigma _{i1}}^2 = \lambda _{i1}$, where $\beta$ is a $p \times 1$ vector of regression parameters. Then, for $j=2,\ldots ,n_i$, the *conditional* distribution of $Y_{ij}$ given $Y_{ij-1}$ is Poisson with conditional mean $\text{ E }\left( Y_{ij} | Y_{ij-1} \right)$ = ${\lambda _{ij}}^{*}$ given by Eq. 3, with

5 $$\begin{aligned} \mu _{ij} = \lambda _{ij} = \text{ exp }\left( x_{ij}^\prime \beta \right) , \end{aligned}$$

and

6 $$\begin{aligned} {\sigma _{ij}}^2 = \lambda _{ij}/\left( 1 - \alpha ^2\right) , \end{aligned}$$

for $j = 2, \ldots ,n_i$ and $i=1,\ldots ,m$. The $Y_{ij}$ are over-dispersed relative to the Poisson distribution if $j \ge 2$ and $\alpha \ne 0,$ because in this case ${\sigma _{ij}}^2 = \phi \lambda _{ij}$, where $\phi$ > 1.

The likelihood can then be expressed as

$$\begin{aligned} L(\beta ,\alpha )= & {} \prod _{i=1}^{m} \text{ P }\left( Y_{i1}=y_{i1}\right) \text{ P }\left( Y_{i2}=y_{i2}| Y_{i1}=y_{i1}\right) \cdots \text{ P }\left( Y_{in_i}=y_{in_i}| Y_{in-1}=y_{in-1}\right) \\= & {} \prod _{i=1}^{m} \frac{\text{ exp }\left( -\lambda _{i1}\right) {\lambda _{i1}}^{y_{i1}}}{y_{i1}!} \prod _{j=2}^{n_i} \frac{\text{ exp } \left( -{\lambda _{ij}}^{*}\right) {\left( {\lambda _{ij}}^{*}\right) }^{y_{ij}}}{y_{ij}!} \\= & {} \prod _{i=1}^{m} \text{ exp }\left( y_{i1} \text{ ln }\left( \lambda _{i1}\right) -\lambda _{i1} - \text{ ln }\left( y_{i1}!\right) \right) \prod _{j=2}^{n_i} \text{ exp }\left( y_{ij} \text{ ln }\left( {\lambda _{ij}}^{*}\right) -{\lambda _{ij}}^{*} - \text{ ln }\left( y_{ij}!\right) \right) . \end{aligned}$$

Taking the natural logarithm then yields the log-likelihood,

$$\begin{aligned} \text{ ln }\left( L(\beta ,\alpha ) \right)= & {} \sum _{i=1}^{m} \left( y_{i1} \theta _{i1} - \text{ exp }\left( \theta _{i1}\right) - \text{ ln }\left( y_{i1}!\right) \right) + \sum _{j=2}^{n_i} \left( y_{ij} {\theta _{ij}}^* - \text{ exp }\left( {\theta _{ij}}^*\right) - \text{ ln }\left( y_{ij}!\right) \right) , \end{aligned}$$

where $$\theta _{i1} = \text{ ln }(\lambda _{i1}) = x_{i1}^\prime \beta $$ and $${\theta _{ij}}^* = \text{ ln }({\lambda _{ij}}^*).$$

The following constraints must be satisfied in order for the constructed likelihood to be valid: (1) $\lambda _{ij} > 0$ $(j=1, \ldots , n_i)$; (2) $-1< \alpha < 1$ $(j=2, \ldots , n_i)$, in order to achieve a positive-definite correlation matrix; and (3) $\lambda _{ij} - \alpha \sigma _{ij}/\sigma _{ij-1}(\lambda _{ij-1}) > 0$ $(j=2, \ldots , n_i)$ (Guerra and Shults 2014).

### Likelihood equations

To obtain maximum likelihood estimates of $\beta$ and $\alpha$, we need to obtain simultaneous solutions to the following estimating equations for $\beta$ and $\alpha$, respectively:

7 $$\begin{aligned} \frac{ \partial \text{ ln }\left( L(\beta ,\alpha ) \right) }{\partial \beta }&=  \sum _{i=1}^{m} \left( y_{i1} - \text{ exp }\left( \theta _{i1}\right) \right) \frac{\partial \theta _{i1}}{\partial \beta } + \sum _{j=2}^{n_i} \left( y_{ij} - \text{ exp }\left( {\theta _{ij}}^*\right) \right) \frac{\partial {\theta _{ij}}^*}{\partial \beta } \\ &= 0 \nonumber \end{aligned}$$

and

8 $$\begin{aligned} \frac{ \partial \text{ ln }\left( L(\beta ,\alpha ) \right) }{\partial \alpha }&=  \sum _{i=1}^{m} \left( y_{i1} - \text{ exp }\left( \theta _{i1}\right) \right) \frac{\partial \theta _{i1}}{\partial \alpha } + \sum _{j=2}^{n_i} \left( y_{ij} - \text{ exp }\left( {\theta _{ij}}^*\right) \right) \frac{\partial {\theta _{ij}}^*}{\partial \alpha } \\ &= 0. \nonumber \end{aligned}$$

The derivatives are provided in Appendix A of the longer, working version of this paper (Gamerman et al. 2016 at http://biostats.bepress.com/upennbiostat/art45). We maximized the likelihood using an adaptive barrier algorithm as implemented in the constrOptim function in R (R Core Team 2014). We applied the Broyden-Fletcher-Goldfarb-Shanno (BFGS) optimization method by Broyden (1970), Fletcher (1970), Goldfarb (1970), and Shanno (1970), Shanno and Kettler (1970), which is implemented in constrOptim when the gradient is provided.

The following algorithm summarizes our estimation procedure for a particular model:

1. Choose initial estimates (starting values) of $\alpha$ and $\beta$. Starting values can be obtained using GEE to fit a Poisson model with an AR(1) correlation structure; however, we should check that the starting values satisfy the constraints ("Poisson Likelihood"). If the estimates violate the constraints, change the starting values by choosing a value for $\alpha$ that is closer to zero or by applying Poisson regression, which is equivalent to assuming that $\alpha = 0$.
2. Obtain solutions to the likelihood Eqs. 7 and 8 using the adaptive barrier algorithm that is implemented in the R package constrOptim. R code for the log likelihood function and for the gradient function, both of which are implemented in the Application, are provided in Appendix B of Gamerman et al. 2016 (at http://biostats.bepress.com/upennbiostat/art45).

### Asymptotic distribution of the estimators

If the model is correctly satisfied and standard regularity conditions are satisfied, the ML approach described here will yield estimates that are consistent and asymptotically normal. Let $\theta = (\beta , \alpha )^T$ and the maximum likelihood estimators ${\hat{\theta }} = ({\hat{\beta }}, {\hat{\alpha }})^T$. We estimated the asymptotic covariance matrix of ${\hat{\theta }}$ with the inverse of the observed information, $(i({\hat{\theta }}))^{-1},$ that we estimated using the inverse of the negative Hessian matrix, which is defined and implemented in Appendices A and B, respectively, of Gamerman et al. (2016 at http://biostats.bepress.com/upennbiostat/art45).

## Application

### Doctor visits data

Here we consider an analysis of a subset of data from the German Socio-Economic Panel data (Winkelmann 2004) that we obtained within Stata (http://www.stata-press.com/data/r14/drvisits) and then exported for analysis in R (StataCorp 2013). Here we compare the results of an analysis using the proposed ML approach with the results obtained using Poisson regression and GEE.

The goal of the analysis was to assess the impact of the 1997 health reform on the reduction of government expenditures. A sample of 1518 women who were employed full time in the year before or after the reform was implemented were evaluated. The outcome we considered was the self-reported number of doctor visits in the three months prior to the interview. The main covariate of interest was an indicator variable that took value 1 if the interview took place after the reform was implemented (or took value 0 otherwwise). Additional covariate information was available on each participant’s age, education, marital status, self-reported health status, and the logarithm of household income. Of the 1518 women in the dataset, 709 were interviewed both before and after the reform was implemented; 391 were only interviewed before; and 418 were only interviewed after the reform went into effect. This resulted in a total of 2227 observations available for the analysis.

We assumed Eq. 5 with the following linear predictor:

$$\begin{aligned} x_{ij} = \beta _0 + \beta _1 x_{ ij1} + \beta _2 x_{ij2} + \beta _3 x_{ij3} + \beta _4 x_{ij4} + \beta _5 x_{ij5} + \beta _6 x_{ ij6}, \end{aligned}$$

where $x_{ij1}$ was the indicator variable for health care reform (1 if after implementation; 0 if before), $x_{ij2}$ was age in years, $x_{ij3}$ was education in years, $x_{ij4}$ was marital status (1 if married; 0 if not married), $x_{ij5}$ was self-reported health status (1 if bad; 0 if not bad), and $x_{ij6}$ was the logarithm of household income.

We first fit the above model using Poisson regression as implemented in the glm function in R; the results are provided in Table 1. Among women with the same household income, marital status, self-reported health, and education, there was a reduction in the log count of doctor visits of 0.140 after health care reform was implemented ($p<0.0001$).

Next, we used the geeglm function in R to implement GEE with an assumed AR(1) working correlation structure; the results are shown in Table 1. As for Poisson regression, there was a significant reduction in the log count of doctor visits (${\hat{\beta }}_1 = -0.123, p=0.0202$). The estimated correlation parameter was 0.213.

When we fit the GEE model we assumed that the scalar parameters $\phi$ = 1 $\forall$ *i*, *j*. After fitting GEE, we assessed the adequacy of this assumption by obtaining an estimate of $\phi$ based on the final GEE estimates of $\beta$:

$$\begin{aligned} {\widehat{\phi }} = \frac{1}{m} \sum _{i=1}^m \frac{Z_i\left( \widehat{{ \varvec{\beta }}}\right) 'Z_i \left( \widehat{{ \varvec{\beta }}}\right) }{n_i}, \end{aligned}$$

where $Z_i(\widehat{{ \varvec{\beta }}})$ is the $n_i \times 1$ vector of Pearson residuals $z_{ij}(\widehat{{ \varvec{\beta }}})$ with $z_{ij}(\widehat{{ \varvec{\beta }}})$ = $\frac{ y_{ij} - \widehat{\lambda _{ij}}}{\sqrt{\widehat{\lambda _{ij}}}}$. The estimated $\phi$ was ${\hat{\phi }} = 4.33,$ which is much greater than 1 and was therefore suggestive of over-dispersion in the data.

Lastly, we fit the proposed ML approach using the algorithm for estimation described in "Likelihood equations" section. We obtained starting values for our approach using GEE, after first confirming that ${\hat{\alpha }}$ satisfied the necessary constraint to guarantee a valid parent distribution, which in this case was ${\hat{\alpha }} < 0.4494$.

Table 1 shows the results for the ML approach. The estimated correlation parameter was 0.313 with a 95% confidence interval of (0.272, 0.354). After adjusting for the correlation among the counts of doctors visits, for over-dispersion, and for the other covariates, we again found that there was a significant impact of initiation of health care reform on the number of doctor visits (${\hat{\beta }}_1 =-0.113, p<0.0001$).

**Table 1:** Estimated parameters from the ML, GEE, and Poisson models in the analysis of the doctor visits data

Parameter	Estimate	SE	Wald	Pr(>|W|)
*Coefficients*				
*ML approach* ($AIC = 11707; BIC = 11{,}750$)				
(Intercept)	−0.461	0.2811	2.69	0.1008
Reform	−0.113	0.0241	21.99	<0.0001
Age	0.005	0.0014	12.22	0.0005
Education	−0.008	0.0064	1.54	0.2153
Marital status	0.026	0.0294	0.75	0.3855
Health status	1.100	0.0313	1238.28	<0.0001
Log income	0.150	0.0376	15.83	<0.0001
Correlation parameters				
Alpha	0.313	0.0208		
GEE approach				
(Intercept)	−0.381	0.5766	0.44	0.5083
Reform	−0.123	0.0529	5.40	0.0202
Age	0.005	0.0033	2.44	0.1182
Education	−0.009	0.0118	0.61	0.4349
Marital status	0.038	0.0698	0.30	0.5822
Health status	1.105	0.0873	160.23	<0.0001
Log income	0.139	0.0798	3.05	0.0809
Correlation parameters				
Alpha	0.213	0.0238		

Parameter	Estimate	SE	z value	Pr(>|z|)
*Coefficients*				
Poisson regression ($AIC = 11,899; BIC = 11,942$)				
(Intercept)	−0.414	0.2691	−1.54	0.1242
Reform	−0.140	0.0265	−5.28	<0.0001
Age	0.004	0.0013	3.35	0.0008
Education	−0.011	0.0060	−1.78	0.0743
Marital status	0.041	0.0278	1.49	0.1375
Health status	1.133	0.0303	37.40	<0.0001
Log Income	0.149	0.0360	4.14	<0.0001

Overall, the parameter estimates were similar for the proposed ML approach, GEE, and the Poisson regression. While the impact of age was similar across the approaches, it was significant in both the ML and Poisson approaches but not significant in the GEE model (ML $p = 0.0005$, GEE $p = 0.1182$, and Poisson $p = 0.0008$). Similarly, the logarithm of household income was significant in both the ML and Poisson approaches but not significant in the GEE model (ML $p<0.0001$, GEE $p = 0.0809$, and Poisson $p<0.0001$).

With estimates of the log-likelihood for Poisson regression and the proposed ML approach, it was possible to calculate the AIC and BIC criteria as measures of the relative quality of the models for this set of data. Both BIC and AIC incorporate a penalty term for the number of parameters used in the model because it is possible to increase the numerical value of the likelihood solely by including additional parameters in the model, which may result in over-fitting the model to the data. This penalty term is larger in the BIC as compared to the AIC. For the Poisson regression model, the AIC and BIC values were 11,899 and 11,939, which were both greater than the AIC and BIC values for the ML approach (AIC = 11,707 and BIC = 11,746), which indicates that the ML approach had improved model fit over Poisson regression.

### Epilepsy seizure data

Here we implement the proposed ML method and GEE for analysis of the epilepsy seizure data (Thall and Vail 1990; Farewell and Farewell 2013) that is available as part of the MASS package in R (Venables and Ripley 2002). We assumed Eq. 5 with the following linear predictor:

9 $$\begin{aligned} x_{ij}'\beta = \beta _0 + \beta _1 x_{ij1}+ \beta _2 x_{ij2}+ \beta _3 x_{ij3} + \beta _4 x_{ij4}, \end{aligned}$$

where $x_{ij1}$ represents an indicator for treatment, $x_{ij2}$ represents baseline seizure count (number of seizures in the 3 month time period prior to the start of the study), $x_{ij3}$ represents subject age in years, and $x_{ij4}$ represents two-week time period (coded as 1, 2, 3, 4). We initially included a time period by treatment interaction term, but the interaction term was not significant for the proposed approach or for GEE (both p-values $> 0.05$); we therefore initially focused on the simpler model 9 for this demonstration.

Table 2 shows the sample mean and variance of seizure counts at baseline and the four subsequent two-week periods (denoted as Y1 through Y4) for the placebo and drug groups for the seizure counts; it also displays the sample mean and variance of age at baseline. From the table, the sample variance for the outcome variables, Y1 through Y4, were greater than their respective means, which suggested that there was over-dispersion in the seizure counts.

**Table 2:** Mean and variance for the placebo and treatment groups

Variable	Placebo$^{\dagger }$	Drug$^{\dagger }$	Total$^{\dagger }$
	(n = 28)	(n = 31)	(n = 59)
Y1	9.86 (102.8)	8.58 (332.7)	8.95 (220.2)
Y2	8.29 (66.6)	8.42 (140.7)	8.36 (103.8)
Y3	8.79 (215.2)	8.13 (192.9)	8.44 (200.2)
Y4	7.96 (58.2)	6.71 (126.8)	7.31 ( 93.1)
Baseline	30.79 (681.2)	31.61 (782.9)	31.22 (722.5)
Age	29.00 (36.0)	27.74 (43.6)	28.34 (39.7)

$^{\dagger }$ Values in the table represent the mean (variance)

Table 3 shows the results of the analysis. The estimates were similar for the proposed ML method and GEE. The estimate of treatment was negative for both approaches, which suggested that the number of seizures was lower for subjects in the treatment group. However, treatment only differed significantly from 0 for the proposed ML approach ($p =0.0124$ for ML versus $p=0.3014$ for GEE). In addition, time period only differed significantly from 0 for the proposed ML approach ($p =0.0032$ for ML versus $p=0.0580$ for GEE).

**Table 3:** Estimated parameters from the GEE and ML approaches for analysis of the epilepsy data when Period is included in the models

Parameter	Estimate	SE	Wald	Pr(>|W|)
ML approach ($AIC = 1566; BIC = 1579$)				
*Coefficients*				
(Intercept)	0.6569	0.1958	11.26	0.0008
Treatment	−0.1668	0.0667	6.26	0.0124
Baseline	0.0232	0.0007	1111.24	<0.0001
Age	0.0238	0.0056	17.94	<0.0001
Period	−0.0634	0.0215	8.72	0.0032
Correlation parameters				
Alpha	0.416	0.0334		
GEE approach				
(Intercept)	0.5855	0.3491	2.81	0.0936
Treatment	−0.1642	0.1589	1.07	0.3014
Baseline	0.0232	0.0012	350.97	<0.0001
Age	0.0263	0.0118	4.95	0.0261
Period	−0.0644	0.0340	3.59	0.0580
Correlation parameters				
Alpha	0.551	0.0656		

The likelihood ratio test of the hypothesis that the regression parameter for time period is 0 also suggested that time period should be retained in the model for the proposed ML approach (*p* = 0.0030.) However, since the GEE analysis suggested that time period might not be important, we removed time period from the model for both GEE and the proposed ML approach. As shown in Table 4, treatment differed significantly from 0 for the proposed ML approach, but was not significant for GEE ($p=0.0121$ for ML versus $p=0.2977$ for GEE).

**Table 4:** Estimated parameters from the GEE and ML approaches for analysis of the epilepsy data when period is not included in the models

Parameter	Estimate	SE	Wald	Pr(>|W|)
*Coefficients*				
ML approach ($AIC = 1573; BIC = 1583$)				
(Intercept)	0.5072	0.1894	7.17	0.0074
Treatment	−0.1673	0.0667	6.30	0.0121
Baseline	0.0232	0.0007	1113.57	<.0001
Age	0.0238	0.0056	17.99	<.0001
Correlation parameters				
Alpha	0.423	0.0342		
GEE approach				
(Intercept)	0.4467	0.3621	1.52	0.2174
Treatment	−0.1659	0.1593	1.09	0.2977
Baseline	0.0232	0.0012	353.32	<.0001
Age	0.0258	0.0117	4.86	0.0275
Correlation parameters				
Alpha	0.544	0.0639		

We next compared the AIC and BIC for the models that included and excluded time period. As shown in the Tables, both the AIC and BIC values were smaller for the larger model that included time period. The respective AIC and BIC values were 1566 and 1579 for the larger model, versus 1573 and 1583 for the smaller model. The AIC and BIC values indicated that the fit was superior for the larger model, which lent additional support for the larger model with its significant treatment and time period effects.

## Simulation studies

In the previous section we identified significant treatment effects for the proposed ML approach that were not observed for GEE. Since the results depended on choice of approach, it was of interest to compare the performance of the methods for finite samples. We therefore performed simulations to assess the properties of the estimators of $\alpha$ and $\beta$ for the proposed ML approach and GEE.

### Set-up

We compared the performance of the ML and GEE estimators for

10 $$\begin{aligned} x_{ij}'\beta = \beta _0 + \beta _1 x_{ij1}+ \beta _2 x_{ij2}+ \beta _3 x_{ij3}, \end{aligned}$$

where the $x_{ijk}$ were defined in the previous section.

The results shown here are based on $R=1000$ simulation runs, equal group sizes *m*/2, ${\beta } = (0.4467, -0.1659, 0.0232, 0.0258)'$ (based on GEE estimates), and $n_i$ = 4 measurements per subject. For this scenario, the correlation must satisfy the constraint $\alpha < 0.707$ (see "Poisson likelihood") to ensure the existence of a valid parent distribution. We specified values of $\alpha \in \{ 0.2, 0.4, 0.6, 0.7 \}.$

Covariates were simulated based on the observed epilepsy seizure data in the previous section. Treatment was specified as present (equal to 1) for one group and as absent (equal to 0) for the other group. Baseline seizure count was simulated from a Poisson distribution with a random seed and mean $= 31.22$ based on the mean baseline age from the epilepsy data. Similarly, age was simulated from a normal distribution based on the epilepsy data for which the minimum age was 18, the mean was 28.3, and the standard deviation was 6.261. Simulated age values below 18 were discarded and the next simulated age value was assigned. Age was then rounded to a whole number, as it was recorded in the epilepsy data.

The approach proposed by Guerra and Shults (2014) was used to simulate the correlated Poisson seizure counts with specified means, over-dispersion, and AR(1) correlation structure.

### Assessments

We wrote code in R to evaluate mean square error (MSE), percent bias, small sample efficiency, and 95% coverage probabilities using the observed information matrix. The mean square error (MSE) for estimator ${\hat{\theta }}$ is defined as

$$\begin{aligned} \frac{1}{R}\sum _{i=1}^R \left( \theta - {\hat{\theta }}_i\right) ^2, \end{aligned}$$

where $\theta$ is the true value. The percent bias for estimator ${\hat{\theta }}$ is defined as

$$\begin{aligned} \left\{ \frac{1}{R}\sum _{i=1}^R \left( \theta - {\hat{\theta }}_i\right) /\theta \right\} *100. \end{aligned}$$

Lastly, to evaluate the coverage probabilities, a 95% confidence interval was computed for each parameter estimate within each simulation run. The coverage probabilities represent the proportion of the R simulation runs in which the true parameter fell within the 95% confidence bounds. GEE coverage probabilities were computed similarly using the naïve variance estimates obtained from geeglm in R.

### Results

Table 5 displays the MSE and Table 6 displays the percent bias for the simulations. For the ML method, the MSE for ${\hat{\beta }}$ and ${\hat{\alpha }}$ and the percent bias for ${\hat{\alpha }}$ decreased as *m* increased.

**Table 5:** Small sample efficiencies for evaluating the AR(1) correlation structure for varying values of $\alpha$ and sample size per group

*m*	$\alpha$	R*	${\hat{\beta }}_0$	${\hat{\beta }}_1^{[1]}$	${\hat{\beta }}_2^{[2]}$	${\hat{\beta }}_3^{[2]}$	${\hat{\alpha }}^{[1]}$
Mean squared error using ML							
60	0.2	1000	0.056	0.355	0.297	0.291	0.609
	0.4	1000	0.088	0.503	0.427	0.445	0.505
	0.6	1000	0.127	0.803	0.642	0.619	0.308
	0.7	998	0.132	0.908	0.716	0.656	0.171
120	0.2	1000	0.029	0.176	0.138	0.137	0.305
	0.4	1000	0.040	0.254	0.203	0.194	0.236
	0.6	1000	0.054	0.381	0.291	0.294	0.124
	0.7	1000	0.067	0.489	0.349	0.325	0.067
300	0.2	1000	0.010	0.071	0.057	0.054	0.111
	0.4	1000	0.016	0.101	0.084	0.078	0.080
	0.6	1000	0.025	0.153	0.121	0.118	0.047
	0.7	1000	0.029	0.174	0.144	0.140	0.023
Mean squared error using GEE							
60	0.2	1000	0.057	0.355	0.300	0.290	0.668
	0.4	1000	0.089	0.516	0.427	0.450	0.701
	0.6	1000	0.137	0.852	0.703	0.653	0.571
	0.7	1000	0.160	1.133	0.883	0.795	0.424
120	0.2	1000	0.029	0.176	0.139	0.138	0.340
	0.4	1000	0.040	0.260	0.204	0.198	0.334
	0.6	1000	0.062	0.415	0.327	0.325	0.240
	0.7	1000	0.083	0.595	0.435	0.402	0.178
300	0.2	1000	0.010	0.072	0.058	0.054	0.128
	0.4	1000	0.017	0.103	0.085	0.079	0.124
	0.6	1000	0.027	0.162	0.132	0.129	0.093
	0.7	1000	0.036	0.211	0.182	0.176	0.066

The true correlation structure is AR(1)

There are equal sample sizes of $\frac{m}{2}$ per group and $\beta = (\beta _0, \beta _{drug}, \beta _{baseline}, \beta _{age})' = (0.4467, -0.1659, 0.0232, 0.0258)'$; [1] True value by a factor of $10^2$; [2] True value by a factor of $10^4$

**Table 6:** Percent bias for evaluating the AR(1) correlation structure for varying values of $\alpha$ and sample size per group

*m*	$\alpha$	R*	${\hat{\beta }}_0$	${\hat{\beta }}_1$	${\hat{\beta }}_2$	${\hat{\beta }}_3$	${\hat{\alpha }}$
Percent bias using ML							
60	0.2	1000	2.57	0.53	−0.61	−0.53	9.41
	0.4	1000	6.33	−0.42	−1.15	−2.31	5.15
	0.6	1000	1.95	0.05	−0.95	0.27	2.65
	0.7	998	−2.21	2.71	0.72	1.21	0.69
120	0.2	1000	−0.04	0.14	0.07	0.20	5.30
	0.4	1000	2.25	−0.52	−0.57	−0.65	2.74
	0.6	1000	0.43	−0.79	0.08	−0.13	1.39
	0.7	1000	2.00	0.15	−0.01	−1.04	0.17
300	0.2	1000	0.68	−0.18	−0.57	0.22	2.83
	0.4	1000	0.85	−0.29	−0.16	−0.25	1.31
	0.6	1000	1.91	−0.38	−0.30	−0.75	0.53
	0.7	1000	1.47	−0.29	−0.14	−0.63	−0.03
Percent bias using GEE							
60	0.2	1000	2.48	0.54	−0.60	−0.49	10.94
	0.4	1000	6.26	−0.34	−1.10	−2.29	6.06
	0.6	1000	1.88	0.64	−0.90	0.45	4.86
	0.7	1000	0.60	1.87	−0.28	0.84	4.60
120	0.2	1000	−0.22	0.07	0.13	0.24	6.19
	0.4	1000	1.95	−0.51	−0.40	−0.64	2.89
	0.6	1000	0.16	−1.05	0.34	−0.21	2.18
	0.7	1000	1.74	−0.22	0.14	−0.83	2.55
300	0.2	1000	0.65	−0.23	−0.57	0.23	2.87
	0.4	1000	0.72	−0.32	−0.15	−0.18	0.98
	0.6	1000	2.03	−0.33	−0.23	−0.88	0.86
	0.7	1000	2.83	−0.20	−0.58	−0.91	1.64

The true correlation structure is AR(1)

There are equal sample sizes of $\frac{m}{2}$ per group and $\beta$ = $(\beta _0, \beta _{drug}, \beta _{baseline}, \beta _{age})' = (0.4467, -0.1659, 0.0232, 0.0258)'$

As compared to GEE, the ML approach had lower MSE and percent bias for all sample sizes for ${\hat{\alpha }}$. For ${\hat{\beta }}$, the percent bias was similar for ML and GEE; however, the MSE was slightly smaller for ML than for GEE. For scenarios with high correlation ($\alpha =0.6$ or 0.7), the intercept and treatment estimates, ${\hat{\beta }}_0$ and ${\hat{\beta }}_1$, had smaller MSE and percent bias for the proposed ML approach than for GEE, for all samples sizes.

Table 7 then displays the estimated coverage probabilities. With respect to ${\widehat{\beta }}$, the coverage probabilities were similar for the ML and GEE approach and were close to the nominal 95% level. With respect to ${\widehat{\alpha }}$, the ML approach model-based coverage probabilities were close to the nominal 95%, which outperformed the GEE approach, whose model-based coverage probabilities were below the nominal 95% level. Coverage probabilities for $\alpha$ were better for the ML based approach than GEE across all sample sizes and correlations ($\alpha = 0.2, 0.4, 0.6, 0.7$).

**Table 7:** Coverage probabilities for the ML and GEE approaches with the AR(1) correlation structure for varying values of $\alpha$ and sample size per group

*m*	$\alpha$	Method	R	Coverage Probability				
				${\hat{\beta }}_0$	${\hat{\beta }}_1$	${\hat{\beta }}_2$	${\hat{\beta }}_3$	${\hat{\alpha }}$
60	0.2	ML	1000	94.7	95.2	95.5	95.5	93.8
		GEE	1000	94.4	95.0	94.8	95.1	91.1
	0.4	ML	1000	93.8	94.6	95.9	93.0	94.6
		GEE	1000	93.2	94.3	95.5	92.7	86.1
	0.6	ML	1000	93.8	93.9	94.3	94.0	93.4
		GEE	1000	94.1	93.6	95.1	93.1	83.2
	0.7	ML	998	95.4	95.3	95.4	95.5	92.3
		GEE	1000	95.0	94.9	94.0	95.7	84.6
120	0.2	ML	1000	94.7	95.2	95.2	94.8	92.9
		GEE	1000	94.2	95.1	94.9	94.5	91.3
	0.4	ML	1000	95.1	96.1	95.6	94.7	95.1
		GEE	1000	95.2	96.0	95.5	94.5	85.4
	0.6	ML	1000	95.9	94.5	95.3	94.9	95.5
		GEE	1000	95.5	95.5	95.5	94.9	84.5
	0.7	ML	1000	95.3	94.2	94.7	96.2	92.9
		GEE	1000	95.3	94.2	95.0	95.9	87.2
300	0.2	ML	1000	95.2	95.0	94.7	94.7	94.5
		GEE	1000	95.6	95.3	94.8	94.6	91.5
	0.4	ML	1000	93.5	95.4	94.2	93.9	96.5
		GEE	1000	93.7	96.0	94.9	94.3	86.2
	0.6	ML	1000	93.2	95.4	94.9	94.0	95.2
		GEE	1000	93.8	95.6	94.6	94.9	85.9
	0.7	ML	1000	94.5	95.1	94.1	94.4	92.4
		GEE	1000	94.8	95.9	94.6	94.8	88.0

The true correlation structure is AR(1)

There are equal sample sizes of $\frac{m}{2}$ per group and $\beta$ = $(\beta _0, \beta _{drug}, \beta _{baseline}, \beta _{age})' = (0.4467, -0.1659, 0.0232, 0.0258)'$

## Conclusion

We proposed an ML approach for analysis of equally spaced longitudinal count data that accounts for intra-subject correlation of measurements and over-dispersion. Our application of the ML approach and GEE demonstrated that the results of the analysis differed between approaches, with significant treatment differences observed for some models for the ML approach, but not for GEE. The availability of the AIC and BIC criteria for the ML approach was useful for selecting between nested models. The interested reader can replicate our analyses using code in R that we provided in Appendix B of Gamerman et al. (2016) ( at http://biostats.bepress.com/upennbiostat/art45).

Our simulations demonstrated that the ML approach was similar to or slightly outperformed GEE with respect to MSE, bias, and coverage probabilities, especially for higher values of the correlation (for ${\hat{\beta }}$). That the ML approach outperformed GEE for larger values of the correlation was not surprising. We assumed over-dispersion that was induced by $\alpha$ and that was greater for larger values of $\alpha$. For $\alpha = 0$ the assumed models for the marginal means and correlations would have been identical for the ML approach and GEE. That the differences between the two approaches were greatest for larger values for the correlation was therefore to be expected.

Winkelmann (2004) implemented the Poisson model and several other approaches, including random effects and hurdle models. In future work it could be of interest to extend our comparisons with GEE to include some of the other methods considered by Winkelmann (2004). However, in this paper we focused our attention on comparisons with GEE because GEE is widely used and, unlike the methods considered by Winkelmann (2004), GEE allows for correct specification of the true AR(1) correlation structure that was induced by the model we used to simulate our data. Our comparisons with GEE therefore allowed us to assess the impact of correctly modeling the marginal mean, correlation structure, and over-dispersion (our approach), versus correctly modeling the marginal mean and correlation structure, but incorrectly ignoring the over-dispersion (GEE).

There are some limitations to the proposed ML approach that should be acknowledged. First, we assumed that the adjacent correlations on subjects are constant. The Pearson correlations of the residuals from a Poisson regression for the epilepsy data suggested that there was a 9% difference between the smallest and largest adjacent correlations, so that it may be worthwhile to relax the assumption of equal adjacent correlations for this data set. In addition, it may be of interest to consider an exchangeable correlation structure that assumes equality of all pairwise correlations on a subject. It is a limitation of the proposed approach that it cannot implement an exchangeable structure. In addition, although the proposed approach accounts for over-dispersion in the distribution of $Y_{ij}$ for $j=2,\ldots ,n_i$, it assumes that $Y_{i1}$ is distributed as Poisson. The proposed approach therefore does not account for over-dispersion in the first measurements on each subject, which is appropriate when the over-dispersion is induced by the intra-subject correlation of measurements. In addition, as noted by a reviewer, the proposed approach assumes that the degree of overdispersion is directly related to the strength of the adjacent correlation coefficient. It would be interesting to explore how the method performs if there is weak correlation but strong overdispersion, or strong correlation with weak overdispersion.

## References

[CR1] Akaike H (1974). A new look at the statistical model identification. IEEE Trans Autom Control.

[CR2] Broyden CG (1970). The convergence of a class of double-rank minimization algorithms. J Inst Math Appl.

[CR3] Consul PC, Famoye F (1992). Generalized poisson regression model. Commun Stat Theory Methods.

[CR4] Efron B (1992). Poisson overdispersion estimates based on the method of asymmetric maximum likelihood. J Am Stat Assoc.

[CR5] Farewell DM, Farewell VT (2013). Dirichlet negative multinomial regression for overdispersed correlated count data. Biostatistics.

[CR6] Feller W (1968). An introduction to probability theory and its applications.

[CR7] Fitzmaurice G, Davidian M, Verbeke G, Molenberghs G (2008). Longitudinal data analysis.

[CR8] Fletcher R (1970). A new approach to variable metric algorithms. Comput J.

[CR9] Gabriel KR (1962). Ante-dependence analysis of an ordered set of variables. Ann Math Stat.

[CR10] Gamerman V, Guerra M, Shults J (2016) Maximum likelihood based analysis of equally spaced longitudinal count data with specified marginal means, first-order antedependence, and linear conditional expectations. UPenn Biostatistics Working Papers. Working Paper 45. http://biostats.bepress.com/upennbiostat/art45

[CR11] Gardiner JC, Luo Z, Roman LA (2009). Fixed effects, random effects and GEE: what are the differences?. Stat Med.

[CR12] Ghisletta P, Spini D (2004). An introduction to generalized estimating equations and an application to assess selectivity effects in a longitudinal study on very old individuals. J Educ Behav Stat.

[CR13] Goldfarb D (1970). A family of variable metric updates derived by variational means. Math Comput.

[CR14] Guerra MW, Shults J (2014). A note on the simulation of overdispersed random variables with specified marginal means and product correlations. Am Stat.

[CR15] Liang KY, Zeger SL (1986). Longitudinal data analysis using generalized linear models. Biometrika.

[CR16] Molenberghs G, Kenward MG (2010). Semi-parametric marginal models for hierarchical data and their corresponding full models. Comput Stat Data Anal.

[CR17] R Core Team (2014) R: a language and environment for statistical computing. R Foundation for Statistical Computing, Vienna, Austria. http://www.R-project.org/

[CR18] Schwarz G (1978). Estimating the dimension of a model. Ann Stat.

[CR19] Serfling R (2011) Asymptotic relative efficiency in estimation. In International encyclopedia of statistical science. Springer, Berlin, pp 68–72

[CR20] Shanno DF (1970). Conditioning of quasi-Newton methods for function minimization. Math Comput.

[CR21] Shanno DF, Kettler PC (1970). Optimal conditioning of quasi-Newton methods. Math Comput.

[CR22] Thall PF, Vail SC (1990). Some covariance models for longitudinal count data with overdispersion. Biometrics.

[CR23] Venables WN, Ripley BD (2002) Modern applied statistics with S. Fourth Edition

[CR24] Vinod HD (2002) Econometric applications of generalized estimating equations for panel data and extensions to inference. In: Ullah A, Wan ATK, Chaturvedi A (eds) Handbook of applied econometrics, chapter 26. Marcel Dekker, New York, pp 553–574

[CR25] Winkelmann R (2004). Health care reform and the number of doctor visits- an econometric analysis. J Appl Econ.

[CR26] Zimmerman DL, Nuñez-Antón VA (2010). Antedependence models for longitudinal data.

